# Comparison of the Patient Enablement Instrument (PEI) with two single-item measures among Finnish Health care centre patients

**DOI:** 10.1186/s12913-019-4182-2

**Published:** 2019-06-13

**Authors:** Elina Tolvanen, Tuomas H. Koskela, Elise Kosunen

**Affiliations:** 1 Faculty of Medicine and Health Technology, Tampere University, c/o coordinator Leena Kiuru, Arvo Building B, 33014 Tampere, Finland; 2 Pirkkala Municipal Health Centre, Pirkkala, Finland; 3 Science Centre, Pirkanmaa Hospital District, Tampere, Finland; 4 Centre for General Practice, Pirkanmaa Hospital District, Tampere, Finland

**Keywords:** Patient enablement instrument, Single-item measures, Validity, Reliability, Finland

## Abstract

**Background:**

The Patient Enablement Instrument (PEI) is an established patient-reported outcome measure (PROM) that reflects the quality of appointments with general practitioners (GPs). It is a six-item questionnaire administered to the patient immediately after a consultation. The aim of this study was to evaluate whether a single-item measure could replace the PEI when measuring patient enablement among Finnish health care centre patients.

**Methods:**

Two single-item measures, Q1 and Q2, were chosen for comparison with the PEI. Firstly, a pilot study with questionnaire testing and brief interviews with the respondents were performed in order to assess the content validity of the PEI and the single-item measures. Secondly, a questionnaire study after a single appointment with a GP was carried out in three health care centres in Western Finland in order to evaluate the construct and criterion validity of the single-item measures. A telephone interview was performed 2 weeks after the appointment in order to assess the test-retest reliability of the single-item measures. The sensitivity, specificity, and both positive and negative predictive values of Q1 and Q2 were calculated with different PEI score cut-off points.

**Results:**

Altogether 483 patients with a completed PEI were included in the questionnaire study analyses. Altogether 149 and 175 patients had completed Q1 and Q2, respectively, both in the questionnaire and the telephone interview. The correlations between the PEI and Q1 and Q2 were 0.48 and 0.84, respectively. Both the single-item measures had a high sensitivity and a negative predictive value in relation to patients with lower PEI scores. The reliability coefficients were 0.24 for Q1 and 0.76 for Q2. The test-retest values of Q1, Q2, and the PEI were low.

**Conclusions:**

Q2 seems to be a valid and reliable measure of patient enablement. Q1 seems to be less correlated with the PEI, but it also has a high negative predictive value in relation to low enablement scores.

## Background

The patient’s perception of care is a key element when assessing quality of care. Several patient-reported outcome measures (PROMs) have been produced to measure the patient’s perception of care, and more are being developed [1]. PROMs can be disease-specific – evaluating the symptoms and impacts of a specific condition – or generic – tailored to consider general aspects, such as quality of life or severity of pain [2]. Until recently, the use of PROMs was seldom systematic and depended mostly on the interests of individual organisations or doctors [2]. This has also been the case in Finland, where the health care system is about to undergo a major reform [3]. Under these circumstances, new instruments to evaluate different aspects of health care quality are needed.

The form of a PROM can be anything from a single-item measure to a complicated questionnaire [2]. Traditionally, single-item measures are used to measure global concepts, e.g. pain [4], working ability [5], or quality of life [6, 7]. The advantage for single-item measurements is that they are effortless and quick to answer. Furthermore, they require little space on a survey form. It is suggested that single-item measures are appropriate if the concept to be measured is sufficiently specific and unidimensional rather than multidimensional [4, 8].

**Fig. 1:**
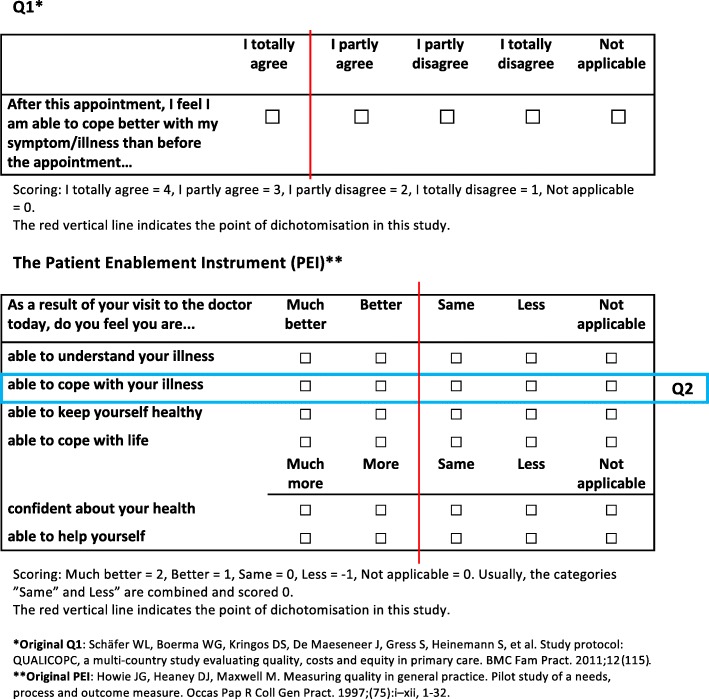
Q1 and the Patient Enablement Instrument (PEI) including Q2

Patient enablement is a concept used to reflect one aspect of health care quality. It is defined as the patient’s ability to understand and cope with illness and life following a consultation with a general practitioner (GP) [9]. This is measured with the Patient Enablement Instrument (PEI), a six-item questionnaire addressed to the patient immediately after the consultation (see Fig. 1). The PEI is suggested to be a good generic PROM [9–11]. Indeed, it is considered “the gold standard” for measuring enablement. This questionnaire has been implemented in several countries, at least in Canada, China (Hong Kong), Croatia, Japan, Poland, United Kingdom and Sweden [9, 12–19].

Based on previous studies, it is clear that several factors are associated with patient enablement. Higher enablement is associated with factors such as longer consultation duration [9, 20], higher patient satisfaction [20, 21], positive experiences of doctor–patient communication [13, 21, 22], and perceptions of the doctor’s empathy [23, 24]. Furthermore, the patient’s poorer state of health [25] and multi-morbidity [13, 23] have been associated with lower enablement. In general, PEI scores seem to vary according to the patients’ ethnic background [12, 13, 26, 27] and between countries [14, 17–19, 28, 29].

Patient enablement could be a potential concept to be measured with a single-item measure. Single-item measures are suggested to be suitable for unidimensional, global concepts [4]. All the PEI’s items are designed to measure one underlying concept, namely patient enablement. Its internal consistency has been shown to be high in previous studies [9, 10, 12, 16, 19], reflecting unidimensionality. To our knowledge, the PEI has not been directly compared to any single-item measure in previous studies.

The aim of this study was to explore whether a single-item measure could replace the PEI in measuring patient enablement among patients at Finnish health care centres. We chose two single-item measures for this comparison. The detailed research objectives were:

1. To determine whether there are correlations between the single-item measures and the PEI (indicating criterion validity);
2. To ascertain what would be the most relevant cut-off point for the PEI score in relation to the single-item measures;
3. To explore the psychometric properties of the single-item measures, focusing on content and construct validity and reliability.

## Methods

### The PEI, the single-item measures Q1 and Q2, and the comparison questions

#### The PEI

The PEI and the single-item measures Q1 and Q2 used in this study are presented in Fig. 1. The PEI questionnaire includes six questions that inquire about the patient’s perceptions of his/her ability to 1) understand his/her problem(s)/illness(s), 2) cope with his/her problem(s)/illness(s), 3) keep him/herself healthy, 4) cope with life, 5) be confident about his/her health, and 6) help him/herself [9].

The scale in the PEI is “much better/more” (2 points), “better/more” (1 point), “same or less” (0 point), and “not applicable” (0 points), leading to a sum score ranging from 0 to 12. This PEI score can be calculated when at least three of the six questions have been answered [9]. There is no clear consensus on what PEI score is considered “good” or “adequate”. PEI score cut-offs of zero [13] or six points [9] have been used, as well as the mean score of the study population at the time [23]. A PEI score of more than six points is suggested to reflect “high” enablement [9].

The PEI questionnaire was formally back-translated into Finnish in 2014 as a part of a larger study [28]. The translation was evaluated by our research team and by a professional translator naive to both versions of the PEI. The translation was concluded to be faithful to the original.

In this study, the PEI was compared to two single-item measures with an almost similar wording but different scales (see Fig. 1):

1. Q1: “After this appointment, I feel I am able to cope better with my symptom/illness than before the appointment.” Possible answers: “I totally agree / I partly agree / I partly disagree / I totally disagree”
2. Q2: “As a result of your visit to the doctor today, do you feel you are able to cope with your illness …” Possible answers: “much better / better / same or less”.

#### Q1

Q1 was included as one of the quality measurements in the Patient Experience questionnaire in the Quality and Costs of Primary Care in Europe (QUALICOPC) study. This question was formed using the PEI questionnaire [30]. Previously, this question has been used to explore factors associated with enablement and coping in Finland [21] and Switzerland [31].

The wording and scoring of Q1 were slightly changed from the original Finnish QUALICOPC questionnaire. Firstly, we changed “health problem/illness” to “symptom/illness”. Secondly, we used a different synonym in Finnish for “coping” in order to achieve better relevance to the Finnish context. In the QUALICOPC study, Q1 had a three-item scale: “no” / “yes” / “don’t know”. We wanted to evaluate whether a four-point Likert-scale would be more relevant, so the items were: “I totally disagree” (1), “I partly disagree” (2), “I partly agree” (3), “I totally agree” (4), and “not applicable”.

#### Q2

Q2 is already part of the PEI questionnaire. The developers of the PEI suggest that this question is one of the three PEI items that have the greatest face validity and are less vulnerable to confounding [13]. In addition, data from previous studies confirm that the three- and six-item measures have a high level of correlation and high internal consistency [11]. Intentionally, the purpose of this study was to explore Q1, but during the research process, it became evident that Q2 had potential properties. Consequently, Q2 was chosen for inclusion in this study. Neither the wording nor the scoring of Q2 was changed.

#### The comparison questions

Some comparison questions were included in the questionnaire in order to assess the construct validity of Q1 and Q2. The comparison questions were “I would recommend this doctor to a friend or a relative”, indicating patient satisfaction; “I benefited from this appointment”, indicating experienced benefit; “I was involved in the decisions made in the appointment”, indicating patient involvement; and “I got adequate instructions to carry on with my care”, indicating instruction evaluation. As with Q1, the same four-point Likert scale was used.

### Study design

The study consisted of three parts:

1. A pilot study that included interviews with patients who filled in the study questionnaires. The purpose of the pilot study was to assess the content validity of PEI (including Q2) and Q1.
2. A questionnaire study with questionnaires (A) before and (B) after the appointment with a GP. Questionnaire A included questions, e.g. about the patient’s self-management and expectations about the consultation, and questionnaire B included the PEI, other assessments of the appointment, and the patient’s demographic information. The purpose of the questionnaire study was to collect quantitative data in order to assess the construct validity, criterion validity, and reliability of Q1 and Q2.
3. A telephone interview was conducted 2 weeks after the appointment to collate information on health service use in the interim period, the PEI, Q1, and comparison questions about patient satisfaction, benefit, involvement, and instruction evaluation. The purpose of the telephone interview was to assess the test-retest reliability of Q1 and Q2.

### Data collection

The study data were collected between February and May 2017. The study was conducted in three municipalities in the Pirkanmaa district in Western Finland: Hämeenkyrö, Pirkkala, and Tampere. The pilot study was performed on a single day when the researcher (ET) recruited patients in the health care centre to fill in the study questionnaires and to participate in a brief interview afterwards. During the data collection period for the actual questionnaire study, the goal was to recruit all patients who had an appointment with a GP at the health centre over a five-day period (Monday to Friday during office hours). The researcher (ET) or research assistants tried to approach everyone who came to the waiting room of the health centre/station during office hours. The exclusion criteria were an age under 18 years, insufficient Finnish skills, and a severity of illness preventing participation in the study. In addition, patients who had an appointment with a GP for maternity or student care were excluded.

All the participants were informed about the study both orally and in writing, and they gave written consent. Paper questionnaires were administered to the participants. Participants who had difficulties with filling in the questionnaire (e.g. due to deteriorated vision) were assisted by the research assistants. All the participants were offered the opportunity to participate in the telephone interview 2 weeks after the appointment. Of the telephone interviewees, those who had had an appointment with a doctor in primary or secondary care in the interim period were excluded from the analyses. This was due to the assumption that potential new interventions in the interim period could affect the later assessments.

### Statistical analyses

All the statistical analyses were performed with IBM SPSS version 25. Descriptive data were used to observe the item variation and discriminative properties of Q1 and Q2. In order to find the most relevant cut-off point for the PEI, cross-tabulations between the PEI and Q1 and Q2 were performed with different PEI cut-offs, and the sensitivity, specificity, and predictive values for Q1 and Q2 were calculated. In terms of construct validity, Spearman correlations between Q1, Q2, the PEI, and the comparison questions were calculated. In terms of reliability, reliability coefficient *r*, mean scores, and Cohen Kappa values for Q1 and Q2 were calculated.

## Results

### Data collection

In the pilot study, 17 of the 32 patients reached were recruited. The mean age of the participants was 59.3 years (range 23–89) and 10 of them (58.8%) were female. In general, the patients accepted the study questionnaires well. The majority of the respondents found the questions important and relevant, and they had no problems when filling out the questionnaires, reflecting the good content validity of both the PEI and Q1.

In the data collection period (17 days), we recruited 546 patients to participate in the study. Of those, 483 had a completed PEI score and were thus included in the analyses. The demographic information of the study sample is presented in Table 1 (see Table 1 attached after the main manuscript). The mean age of the participants was 58.5 years (range 18–97, SD 19.1), and 313 (64.8%) were female. Furthermore, 175 patients who participated in the telephone interview had a completed PEI score and had made no visits to any doctor in the interim period, and thus they were included in the test-retest analyses. Compared to those who did not participate, those who participated in the telephone interview were older, more often retired, had more chronic illnesses, and were more likely to have a higher-level education and to live in a semi-rural location.

**Table 1:** The demographic information of the study sample

	All participants, *n* = 483		Comparison by participation in the telephone interview (test-retest analyses)			
			Patients included in the test-retest analyses, *n* = 175^a^		Patients who did not participate in the telephone interview, *n* = 254	
	Frequency	Percentage	Frequency	Percentage	Frequency	Percentage
Age^b^						
Range	18–97		**19–88**		**18–97**	
Mean (SD)	58.5 (19.1)		**62.2 (17.2)**		**56.2 (20.4)**	
Data missing/NA	17	3.5	7	4.0	9	3.5
Mean PEI score immediately after the appointment						
Mean (SD)	3.78 (3.83)		4.13 (3.95)		3.81 (3.86)	
Sex						
Female	313	64.8	108	61.7	173	68.1
Male	153	32.8	60	34.3	73	28.7
Other	1	0.2	0	0	1	0.4
Data missing/NA	16	3.3	7	4.0	7	2.8
Language						
Finnish	455	94.2	164	93.7	240	94.5
Other	5	1.1	2	1.1	2	0.8
Data missing/NA	23	4.8	9	5.1	12	4.7
Co-habitation						
Single, divorced, widowed	199	41.2	72	41.1	105	41.3
Married, registered partnership, or common-law marriage	267	55.3	96	54.9	140	55.2
Data missing/NA	17	3.5	7	4.0	9	3.5
Education^b^						
No qualifications obtained or primary education (lower-level)	119	24.9	41	23.4	65	25.6
Upper secondary-level education (middle-level)	245	50.7	**80**	**45.7**	**141**	**55.5**
Post-secondary or higher (higher-level)	98	20.3	**47**	**26.9**	**37**	**14.6**
Data missing/NA	21	4.3	7	4.0	11	4.3
Working status^b^						
Working	92	19.0	**21**	**12.0**	**61**	**24.0**
Retired	275	56.9	**112**	**64.0**	**135**	**53.1**
Other (unemployed, student, other)	99	20.5	**34**	**19.4**	**51**	**20.1**
Data missing/NA	17	3.5	8	4.6	7	2.8
State of health (self-assessment)						
Excellent	32	6.6	10	5.7	21	8.3
Good	165	34.2	66	37.5	85	33.5
Fair	171	35.4	60	34.3	85	33.5
Poor	18	3.7	6	3.4	7	2.8
Data missing/NA	97	20.1	33	18.8	56	22.0
Number of chronic illnesses^b^						
No chronic illness	78	16.1	**22**	**12.6**	**48**	**18.9**
1	116	24.0	**38**	**21.7**	**69**	**27.2**
2–3	191	39.5	**80**	**45.7**	**87**	**34.3**
> 3	61	12.6	**26**	**14.9**	**26**	**11.3**
Data missing/NA	37	7.7	9	5.1	24	9.4
Number of reasons for the consultation ^b^						
1	299	61.9	**98**	**56.0**	**170**	**66.9**
> 1	170	35.2	**71**	**40.6**	**77**	**30.3**
Data missing/NA	14	2.9	6	3.4	7	2.8
Reason for the consultation						
Acute	158	32.7	52	29.7	83	32.7
Non-acute	311	64.4	117	66.9	164	64.6
Data missing/NA	14	2.9	6	3.4	7	2.8
Location^b^						
Semi-rural	147	30.4	**58**	**33.1**	**63**	**24.8**
Urban	196	40.6	**78**	**44.6**	**108**	**42.5**
Rural	140	29.0	**39**	**22.3**	**83**	**32.7**

^b^Patients who had not visited a doctor in the interim period and had completed the PEI at the baseline and retest; 26 of these had not completed Q1

^b^Statistically significant difference between groups in the Chi-square test (bolded), missing values excluded from the analyses

### Item distributions of the PEI, Q1, and Q2

The mean PEI score immediately after the appointment was 3.78 (range 0–12, SD 3.83). Altogether 131 of 483 (27.1%) had the floor (0 points) score and 37 (7.7%) the ceiling (12 points) score. There were 16 respondents (3.3%) with at least one item missing.

When considering Q1, 237 patients (49.1%) chose the item “I totally agree” and 149 (30.8%) the item “I partly agree”. The proportions of both disagree options for Q1 were very low (altogether 8.2%), suggesting low discriminative properties. There were 17 (3.5%) missing responses. For the analysis, we decided to dichotomise the answers using “I totally agree” versus “not totally agree” (i.e. the other three options). In addition, while the “not applicable” (NA) values are counted as 0 in the PEI, we combined the NA values (40; 8.3%) with the “not totally agree” group.

With Q2, 98 of 483 patients (20.3%) answered “much better”, 138 (28.6%) answered “better”, and 239 (49.5%) answered “same or less”. Altogether, eight (1.7%) responses were missing. To achieve higher comparability between Q1 and Q2, Q2 was dichotomised as “better or much better” versus “same or less”.

### The sensitivity, specificity, and predictive values of Q1 and Q2 with different PEI score cut-offs

The PEI score cut-offs of zero, three (3.78 being the mean of the study), and six points were used in order to find the most relevant cut-off points in relation to Q1 and Q2. For the different cut-off points, the sensitivity, specificity, and positive and negative predictive values are presented in Table 2. Both Q1 and Q2 had high negative predictive values (95.6 and 98.1%, respectively) with a PEI cut-off score of six points.

**Table 2:** The sensitivity, specificity, and positive and negative predictive values of Q1 and Q2 using different PEI cut-off scores, *n* = 466

PEI cut-off score	Sensitivity (%)		Specificity (%)		Positive predictive value (PPV), (%)		Negative predictive value (NPV), (%)	
	Q1	Q2	Q1	Q2	Q1	Q2	Q1	Q2
Zero points (0 vs 1–12)	86.6	64.8	75.4	100.0	86.9	54.4	41.4	100.0
Three points (0–3 vs 4–12)	69.7	92.0	66.9	81.4	70.0	78.4	68.6	93.3
Six points (0–6 vs 7–12)	90.0	98.1	60.0	63.9	38.4	43.2	95.6	98.1

Sensitivity = the proportion of “true positive” patients, i.e. patients who answered positively to Q1 or Q2 among those who had higher PEI scores

Specificity = the proportion of “true negative” patients, i.e. patients who answered negatively to Q1 or Q2 among those who had lower PEI scores

Positive predictive value = the proportion of patients who actually had a higher PEI score among those who answered positively to Q1 or Q2

Negative predictive value = the proportion of patients who actually had a lower PEI score among those who answered negatively to Q1 or Q2

### Correlations between Q1, Q2, other PEI items, the PEI score, and comparison questions

Spearman correlations between Q1, Q2, other PEI items, the PEI score, and the comparison questions are presented in Table 3. The correlation between Q1 and the PEI items varied from 0.38 (“Keep myself healthy”) to 0.49 (“Cope with illness”). The correlation between Q2 and the other PEI items varied from 0.57 (“Keep confident about my health”) to 0.70 (“Understand illness”). The correlations between Q1 and the PEI score and between Q2 and the PEI score were 0.48 and 0.84, respectively. The correlations between the comparison questions were higher with Q1 (0.31–0.47) than they were with Q2 (0.20–0.29).

**Table 3:** Spearman correlations between Q1, Q2, other PEI items, the PEI score, and the comparison questions; the construct validity of Q1, *n* = 483

PEI item	Q1^a^	Q2^b^
Understand illness	0.40	0.70
Q2: Cope with illness	0.49	1.00
Keep yourself healthy	0.38	0.67
Cope with life	0.43	0.62
Keep confident about your health	0.40	0.57
Help yourself	0.44	0.63
PEI score immediately	0.50	0.84
Comparison question		
I would recommend this doctor to a friend or a relative	0.31	0.20
I benefited from my appointment with this doctor	0.47	0.29
I was involved in the decisions made at the appointment	0.33	0.22
I got adequate instructions to carry on with my care	0.40	0.25

All correlations are statistically significant at the 0.05 level

Note: all variables are non-dichotomised

^a^Q1: “After this appointment, I feel I am able to cope better with my symptom/illness than before the appointment.” Answer options: “I totally agree / I partly agree / I partly disagree / I totally disagree”

^b^Q2: “As a result of your visit to the doctor today, do you feel you are able to cope with illness …” Answer options: “much better / better / same or less”

### The reliability of Q1 and Q2

The reliability of the single-item measures was calculated with the formula $r(xy)=\sqrt{r(xx)\ast r(yy)}$ [8]. In this formula, *r (xy)* is the correlation between variables, *r (xx)* is the reliability of variable x (in this case, the single-item measure Q1 or Q2) and *r (yy)* is the reliability of variable y (in this case, the scale measure PEI). The correlations between Q1 and the PEI and Q2 and the PEI were 0.50 and 0.84, respectively. For the PEI, the Cronbach’s alpha reliability coefficient was 0.93. Using the formula, the reliability was 0.27 for Q1 and 0.76 for Q2.

At the baseline and 2 weeks after the appointment, 149 patients had completed Q1 and 175 patients had completed Q2. In order to evaluate the test-retest reliability of Q1, it was treated as a numeric variable and the means at the baseline and retest were calculated. The mean for Q1 was 3.49 (SD 0.85) at the baseline and 3.03 (SD 0.72) at the retest. The difference between means was statistically significant in the Wilcoxon signed rank test (Z = -5.52, *p* < 0.001). In addition, when treated as categorical variables, the Kappa values measuring total agreement between the baseline and the retest were only 0.21 for Q1 and 0.29 for Q2, confirming the low test-retest reliability of both. The pattern was similar with the PEI score, all other PEI items, and the comparison questions.

## Discussion

This study shows that it is possible to measure patient enablement with a single-item measure. Q2, which is included in the PEI questionnaire, has a strong correlation with the PEI score, a high reliability, and a high sensitivity/negative predictive value with the PEI cut-off scores of three and six. Q1, which is very similar to Q2 but has a different scale, has a high sensitivity and a negative predictive value with a PEI cut-off score of six. However, the correlation with the PEI score and the reliability of Q1 are significantly lower than with Q2. Both Q1 and Q2 seem to measure different concepts, like patient satisfaction or decision involvement. These single-item measures, like the PEI itself, have a low test-retest reliability.

The most notable difference between Q1 and Q2 is the measuring scale; otherwise, they are almost identical. The wording of these measures is very similar. Both questions are transitional, measuring the change in the patient’s perception as a result of the consultation. The different scale is the most probable reason for the modest correlation between Q1 and Q2 and the whole PEI. It seems possible that the four-point Likert scale used in Q1 is too insensitive to detect the change in the patient’s perceptions of coping.

Both Q1 and Q2 seem to identify well the patients with lower enablement scores. Q2 has a high negative predictive value (98.1%) in relation to the PEI with a cut-off of six points, meaning that patients who answered negatively to Q2 had a 98.1% likelihood of having a PEI score of 0–6 points. Q1 has almost as high a negative predictive value, at 95.6%, with the cut-off of six points. When bearing in mind the clinical relevance of this result, we consider simply finding patients with low enablement to be crucial. Such patients might benefit from different interventions or a different health service focus.

Previous studies support the reliability of single-item measures, although their reliability is sometimes questioned [4, 8]. Usually, reliability values > 0.7 are considered adequate [32]. In this study, the reliability of Q2 in relation to the PEI was high, at 0.76, and the reliability of the Q1 was significantly lower, at 0.24. The calculation formula of the reliability coefficient *r* of both measures differs only by the correlation between them and the PEI. Consequently, the notable difference in reliability is caused by the different correlations between Q1 or Q2 and the PEI.

The generally moderate correlations between Q1 and Q2 and the comparison questions suggest the good construct validity of these single-item measures. The comparison questions were more highly correlated with Q1 than with Q2. Both Q1 and Q2 had the highest – albeit moderate – correlations with patient-perceived benefit (0.49 and 0.34, respectively) and instruction evaluation (0.45 and 0.34, respectively). The difference between the correlations may be caused by the different measuring scale. Altogether, the single-item measures seem to measure different concepts from the comparison questions.

The test-retest reliability values of Q1, Q2, and the PEI are low. This indicates that perceptions of enablement seem to diminish after a rather short period of time. This phenomenon was seen also in previous studies [16, 18, 33], as well as with other PROMs [33]. Nevertheless, it is suggested this is not due to the measurement itself, but to a true “dilution” of experience [16, 18]. In addition, the transitional scale could affect the evaluation over time [34, 35]. It could be difficult for the patient to evaluate “whether there had been a change in my perceptions due to an appointment two weeks ago”.

### Strengths and limitations

The theoretical frame supports the idea of using a single-item measure when measuring patient enablement. The concept of enablement is unidimensional [9, 10, 12, 16, 19] and hence suitable for single-item measures. Such single-item measures could save space in questionnaire forms, thus saving time and money for researchers and clinicians. It is also more convenient for the respondent to answer one question instead of six. One limitation of choosing single-item measurement in this study is that Q2 is actually part of the PEI questionnaire. However, we regard that excluding Q2 from the PEI would not reflect the complete measurement and thus be inaccurate. In an comparable situation, the authors came into the similar conclusion when studying different work ability measurements [5].

In this study, all but one respondent in the pilot study found the PEI questions relevant and had no difficulties when filling out the questionnaire form. Nevertheless, the pilot study interviews were made mostly using open questions and the “thinking aloud” technique. The use of more specific and structured questions, as was done in a recent PEI study [34], might have been more appropriate. With this procedure, the non-discriminative scale of Q1 might have been detected earlier. Furthermore, it has been suggested that the PEI could be more vulnerable to hypothesis guessing, and it might lack face validity for some patients [34].

The study sample was altogether satisfactory. It was intended to be the total sample of patients who visited the health care centres during 1 week. During the data collection period, we reached 79.3% of all the patients heading for GP appointments (information derived from the ICT system of the health care centres). This could be regarded as a good result. In addition, although the health care centres were not chosen randomly, they were located in both urban and rural areas with different population structures. Furthermore, the study sample matches fairly well the average users of Finnish health care centres [36], with a slight overrepresentation of female and elderly patients. However, we could not compare the characteristics of participants and non-participants, and a selection bias is therefore possible.

This study presents new information about measuring patient enablement and instrument validity in Finnish primary health care. One limitation of the study is that the validity of the comparison questions has not been evaluated in the Finnish context. Nevertheless, these questions have been used in earlier studies [37, 38]. In general, there are very few PROMs available that have undergone a rigorous assessment for validity and reliability in the Finnish context.

## Conclusions

Patient enablement, regarded as one aspect of quality, could be measured with Q2, a single-item measure. Q2 was extracted from the PEI questionnaire; it has a strong correlation with the PEI score and hence a good reliability. Q2 seems to measure different concepts from, e.g. patient satisfaction or decision involvement, which suggests good construct validity. In addition, Q1, which was developed in the QUALICOPC study, seems to identify well those patients with lower patient enablement scores. Q1 is less correlated with the PEI score compared to Q2. The four-point Likert scale of Q1 is possibly too insensitive. In general, we suggest that both Q1 and Q2 are practicable measures. In particular, Q2 could be used instead of the PEI as a part of an assessment when measuring the quality of clinical performance in GP appointments.

## Data Availability

The datasets used and analysed during the current study are available from the corresponding author upon reasonable request.

## Abbreviations

GP: General practitioner
NA: Not applicable
NPV: Negative predictive value
PEI: Patient Enablement Instrument
PPV: Positive predictive value
PROM: Patient-Reported Outcome Measure
QUALICOPC: Quality and Costs of Primary Care in Europe
